# Randomized crossover trial of amoxapine versus vitamin B_12_ for retrograde ejaculation

**DOI:** 10.1590/S1677-5538.IBJU.2016.0468

**Published:** 2017

**Authors:** Jianlin Hu, Koichi Nagao, Toshihiro Tai, Hideyuki Kobayashi, Koichi Nakajima

**Affiliations:** 1 Department of Urology, Reproduction Center, Toho University School of Medicine, Omori Hospital, Tokyo, Japan;; 2 Department of Reproductive Medicine, Shanghai General Hospital, Shanghai Jiao Tong University School of Medicine, Shanghai, China

**Keywords:** Ejaculation, Drug Therapy, Amoxapine, Infertility, Male

## Abstract

**Objective:**

To compare the efficacy and safety of amoxapine and vitamin B_12_ for treating retrograde ejaculation (RE).

**Materials and Methods:**

Between May 2009 and November 2012, this open-label, randomized, crossover study enrolled 26 men suffering with RE at Department of Reproductive Medicine, Omori Hospital. Patients were randomly allocated into two groups (n=13 each). The amoxapine-B_12_ group received amoxapine (50 mg daily for 4 weeks, orally) followed (after a 1-week washout period) by vitamin B_12_ (500 μg three-times daily for 4 weeks). The B_12_-amoxapine group received the opposite regimen. All patients masturbated to ejaculation at least twice during each treatment period. The primary outcome was antegrade ejaculation of semen, as reported by the patient, on more than one occasion during either treatment period (defined as treatment success). Any adverse events were noted. Success rates were compared between treatments using Fisher’s exact test.

**Results:**

One patient (B_12_-amoxapine group) withdrew for personal reasons (breakdown of marital relations); all other patients completed the study. Overall success rate was 88% (22/25). Success rate was higher for amoxapine than for vitamin B_12_ (80%, 20/25 vs 16%, 4/25; P<0.0001). 18 patients were responsive to amoxapine but not to vitamin B_12_, 2 patients were responsive to vitamin B_12_ but not amoxapine, 2 patients were responsive to both drugs, and 3 patients had no response to either drug. One patient (4%) reported sleepiness and 2 (8%) reported constipation while receiving amoxapine. No adverse events were reported during vitamin B_12_ treatment.

**Conclusions:**

Amoxapine may be an effective, safe and well-tolerated therapy for RE.

## INTRODUCTION

Retrograde ejaculation (RE) is defined as a substantial redirection of seminal fluid from the posterior urethra into the bladder and mainly caused by bladder neck dysfunction (1). Men suffering from RE present with total or sometimes partial absence of semen, despite the sensation of an orgasm, after intercourse or masturbation (2). Current treatment methods are based on two different strategies (3). The first is pharmacologic intervention or surgical management in order to restore antegrade ejaculation by increasing bladder neck tone. The second is urinary sperm retrieval or electroejaculation; this aims to facilitate fertility by obtaining spermatozoa with invasive methods and then applying artificial reproductive technologies.

To date, there has been little guidance on RE management. Nevertheless, pharmacotherapy can be tried as a first-line treatment because it is simple, time-saving, cost-effective and non-invasive. Clinically, imipramine, a tricyclic antidepressant agent (TCA), is commonly used to treat RE due to a variety of causes. However, the overall success rate does not exceed 50%, and adverse effects are reported frequently (4-7). On the other hand, studies of RE therapy, including the use of imipramine medication, have many deficiencies. Most previous investigations of RE therapy are case studies or lack a suitable control, partly because the incidence of RE is not very high. It has been reported that RE accounts for less than 2% of cases of subfertility presenting to a fertility clinic (3). Thus, it can be challenging to obtain a sufficiently large sample size for validate statistical comparisons.

Amoxapine, a tetracyclic antidepressant that is chemically distinct from TCAs, has been reported to result in far fewer adverse events than imipramine in patients treated for depression (8). Amoxapine also selectively blocks neuronal reuptake of norepinephrine and, to a lesser extent, serotonin, and thus upregulates peripheral sympathetic activity to contract the bladder neck; therefore, it also exerts actions that are potentially beneficial in the treatment of RE. Successful treatment of RE with amoxapine has been described in a case report (9). Based on these very limited previous data, we hypothesized that amoxapine would show clinical benefit as a therapy for RE. Therefore, the objective of this randomized crossover trial was to compare the efficacy and safety of amoxapine in the treatment of RE with those of vitamin B_12_, which was used as a negative control.

## MATERIALS AND METHODS

### Study design

This was an open-label, randomized, crossover study comparing the treatment efficacy, adverse effects and tolerability of amoxapine and vitamin B_12_ in the management of RE.

### Patients

The study participants were recruited between May 2009 and November 2012 at the Assisted Reproduction Center, Omori Hospital. The inclusion criteria were: 1) male; 2) aged between 18 to 60 years old; 3) patient reported an absence of antegrade ejaculation but experienced a sensation of orgasm after intercourse or masturbation; and 4) a definitive diagnosis of primary RE or secondary RE (e.g. associated with diabetes mellitus, pelvic surgery or depression) was made on the basis of the detailed medical history, physical examination findings and results of imaging and laboratory investigations, as described below.

The diagnosis of RE was made using a standard protocol. During the initial visit to our reproduction center, all patients underwent a standard evaluation of the male reproductive system according to Japanese reproductive health guidelines (10). A detailed medical history was obtained, and erectile function was evaluated using the International Index of Erectile Function (IIEF-5) questionnaire. We also used ultrasonography to measure the testis volume as well as to evaluate the epididymal and prostatic structures. Furthermore, the presence of the vas deferens and seminal vesicles were confirmed to exclude patients with obstructive problems such as congenital bilateral absence of the vas deferens (CBAVD), ejaculatory duct cyst or dysplasia of seminal vesicles, *etc*. In addition, we conducted laboratory blood testing, including determination of serum concentrations of glucose and sex hormones such as testosterone (T), estradiol (E2), luteinizing hormone (LH), follicle stimulating hormone (FSH) and prolactin (PRL). Examination of semen in the urine was conducted according to World Health Organization guidelines (11). Patients were asked to supply a semen sample by masturbation in our reproduction center after 3 to 5 days of abstinence, and post-masturbatory urine was then collected for analysis. If sperm were found in the post-centrifuge sample (centrifuged at 3000g for 15 minutes and viewed at ^×^200 magnification) and the fructose-resorcinol-hydrochloric acid test was positive (colored), the diagnosis of RE was then confirmed.

Men were excluded if any of the following criteria applied: 1) moderate-to-severe erectile dysfunction (IIEF-5 score ≤ 12); 2) obstructive or non-obstructive azoospermia (absence of vas deferens or absence of sperm in post-masturbatory urine); 3) low sexual desire; 4) serum concentrations of sex hormones not within the normal range; or 5) unable to suspend current pharmacologic treatment for other underlying illnesses (e.g., patients using α1-adrenoceptor antagonists for the treatment of hypertension, angle-closure glaucoma, patients using monoamine oxidase inhibitors, acute and recovery phase of myocardial infarction). Ultimately, 26 patients with confirmed RE were enrolled in this study.

The following baseline demographic and clinical characteristics were recorded for each enrolled patient: age, duration of RE, type of RE (primary or secondary), marital status, and desire for infertility treatment.

This study was approved by the ethics committee of Toho University (approval number 20-105). All patients were informed of the study objectives and design and gave written informed consent before their participation. There were no important changes to the methods after trial commencement.

### Grouping and intervention

**Figure 1:**
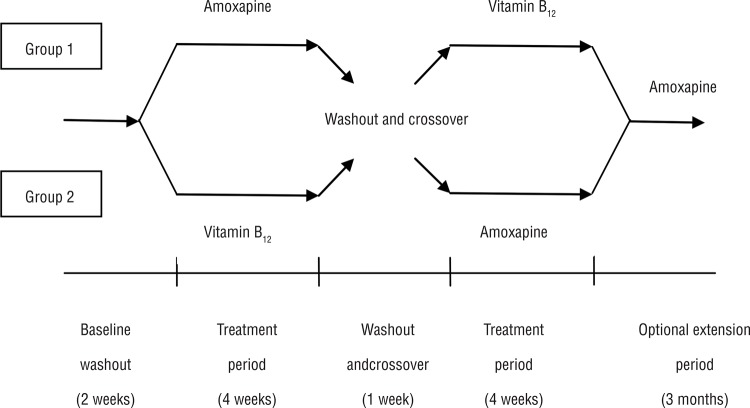
Study design.

The study comprised a 2-week baseline period, two 4-week treatment periods (separated by a 1-week washout period), and an optional 3-month extension period (Figure-1). Briefly, the patients were randomly divided into two groups (n = 13 each) using an allocation sequence generated with a random number table; the random allocation sequence was implemented using sequentially numbered, opaque, sealed envelopes. As this was an open-label study, neither the patient nor investigators were blinded to the treatment used once the allocation had been made. After a 2-week washout period (for drugs used to treat other underlying illnesses, particularly drugs that might affect ejaculation, such as antipsychotics and α_1_-adrenoceptor antagonists), patients allocated to the amoxapine-B_12_ group took amoxapine (Pfizer, Japan) 50 mg daily (orally at bedtime) for 4 weeks. After a 1-week washout period, the medication was changed to vitamin B_12_ (Otsuka, Japan) 500 μg three times daily (orally after meals) for another 4 weeks. Patients in the B_12_-amoxapine group received the opposite regimen.

### Outcome measures

Patients were followed up at the end of each 4-week treatment period. The primary outcome measure was the percentage of patients in which antegrade ejaculation was recovered during the treatment period (‘success rate’). Assessment of this outcome measure was as follows. All patients were instructed to masturbate at least twice during each 4-week treatment period. Recovery of antegrade ejaculation was defined as the ejaculation of white fluid (semen) on more than one occasion during the 4-week treatment period, as reported by the patient at the follow-up consultation. The recovery of antegrade ejaculation was taken to indicate that the pharmacologic intervention had been successful in that particular patient. The success rates were compared between the two medical therapies.

The patients entering the 3-month study extension were followed-up for at least a further 3 months after treatment. During the 3-month study extension, patients with fertility requirements were prescribed amoxapine according to the above regimen and encouraged to attempt timed intercourse at 2 to 0 days before their partner’s ovulation. The occurrence of successful pregnancy during the 3-month extension period was recorded as a secondary outcome measure. If pregnancy did not occur during that time, artificial reproduction techniques such as *in vitro* fertilization (IVF) or intracytoplasmic sperm injection (ICSI) were recommended for consideration.

Any adverse events reported by the patients during the treatment periods were recorded.

There were no changes to the trial outcomes after the trial had commenced.

### Statistical analysis

All analysis was performed using SPSS version 13.0 (SPSS Inc., Chicago, IL USA). The data were analyzed using descriptive statistics and are presented as median, range, frequency or percentage, as appropriate. The success rates were compared between groups using Fisher’s exact test. In all statistical tests, statistical significance was defined as a P value < 0.05.

## RESULTS

**Figure 2:**
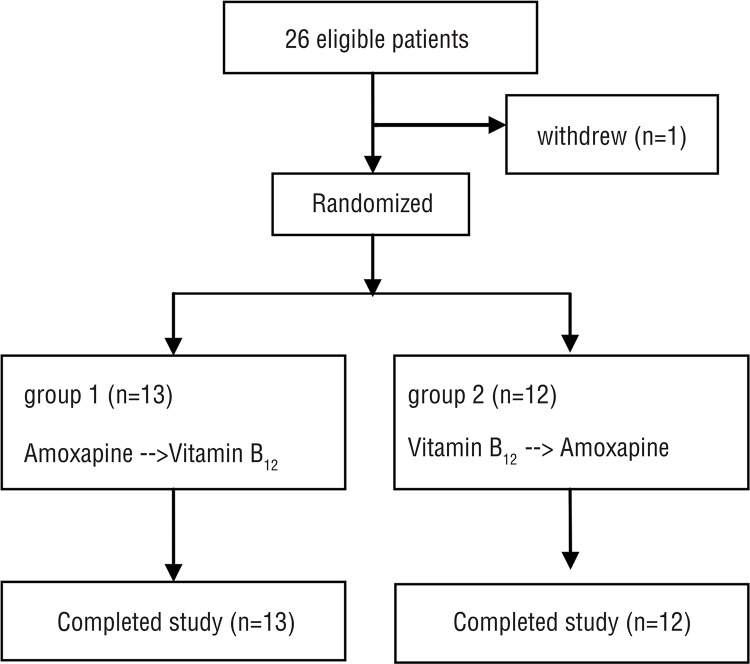
Enrollment and follow up of study subject.

A total of 26 patients were randomized in a 1:1 ratio into the two groups. One patient in the B_12_-amoxapine group withdrew during the first treatment period for personal reasons (breakdown of marital relations and divorce). Ultimately, 25 patients successfully completed the study (13 patients in the amoxapine-B_12_ group and 12 patients in the B_12_-amoxapine group) and were included in the final analysis (Figure-2).

Table-1 shows the demographic characteristics of the patients. The age ranged from 28 to 54 years (median, 40.8 years) while the duration of RE ranged from 2 months to 25 years (median, 4.5 years). Among the 25 patients, 22 (88%) had a previous history of normal ejaculation and were diagnosed as having secondary RE. The cause of RE was diabetes mellitus in 15/22 patients (68.2%), postsurgical complications of radical resection of rectal carcinoma in 6/22 patients (27.3%), and depression in 1/22 patients (4.5%). Due to the absence of a previous history of normal ejaculatory experiences, RE was considered idiopathic or primary in 3/25 patients (12%). A total of 11 patients (44%) were married, 10 of whom (40% of the total) sought treatment for infertility and entered the 3-month period of extended treatment with amoxapine. During follow-up, the wives of two patients (20%, 2/10) became pregnant naturally and the wife of another patient (10%, 1/10) became pregnant by intracytoplasmic sperm injection 6 months later.

**Table 1:** Demographic characteristics of the patients (n = 25).

Variables		
Age (years)	Range	28~54
	Median	40.8
Duration of RE (years)	Range	0.17~25
	Median	4.5
RE type (n)	Primary	3
	Secondary	22
Marriage status (n)	Married	11
	Unmarried	14
Desire for infertility treatment (n)	Yes	10
	No or presently not	15

**Figure 3:**
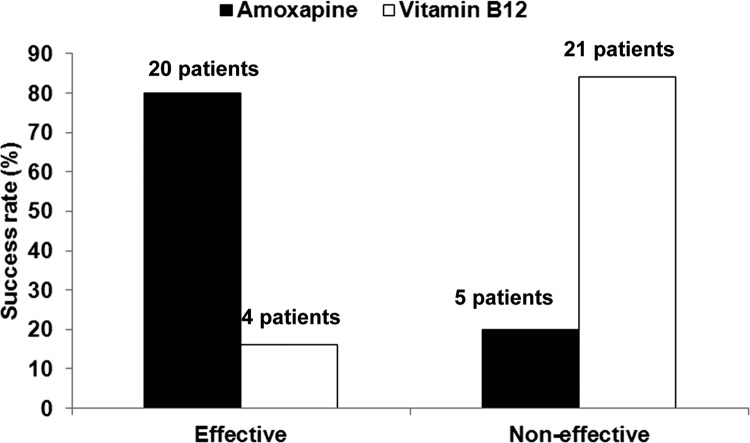
Comparison of success rates for amoxapine and vitamin B12. Fisher’s exact test, P < 0.05.

The treatment outcomes (i.e. numbers of patients in which antegrade ejaculation was recovered during the treatment period) for amoxapine and vitamin B_12_ are shown in Table 2. The overall success rate in all patients was 88% (22/25 patients). The success rate was significantly higher for amoxapine than for vitamin B_12_ (80%, 20/25, 95% CI: 59%-93% vs 16%, 4/25, 95% CI: 5%-36% respectively; P < 0.0001; Figure-3). In total, 18 patients (72%, 18/25, 95% CI: 51%-88%) were responsive to amoxapine (i.e. recovered antegrade ejaculation) but not to vitamin B_12_. In contrast, only 2 patients (8%, 2/25, 95% CI: 1%-26%) were responsive to vitamin B_12_ but not to amoxapine. A further 2 patients (8%, 2/25, 95% CI: 1%-26%) were responsive to both drugs, and 3 patients (12%, 3/25, 95% CI: 3%-31%) had no response to either drug.

**Table 2:** Pharmacologic treatment outcomes.

Cause	n	Efficacy (recovery of antegrade ejaculation)				Total efficacy	

		Amoxapine (n)	Vitamin B12 (n)	Both (n)	Neither (n)	Amoxapine (n)	Vitamin B12 (n)
DM	15	14	0	0	1	14	0
RRRC	6	4	1	1	0	5	2
Depression	1	0	0	1	0	1	1
Idiopathic	3	0	1	0	2	0	1
**Total**	**25**	**18**	**2**	**2**	**3**	**20**	**4**

**DM =** Diabetes mellitus; **RRRC =** Radical resection of rectal carcinoma.

### Adverse events

One patient (4%, 1/25) reported sleepiness and two patients (8%, 2/25) reported constipation while receiving amoxapine. No adverse events were reported during treatment with vitamin B_12_.

## DISCUSSION

The main finding of the present study was that amoxapine showed higher efficacy compared with vitamin B_12_ in the treatment of RE, with a total of 80% (20/25) of all patients responding to the drug. Furthermore, amoxapine was well tolerated, with only mild adverse events reported in a small proportion of patients (12%, 3/25). Therefore, low-dose amoxapine may be safe and effective for the management of RE.

Amoxapine is an N-demethylated dibenzoxazepine and has antidepressant properties that resemble those of imipramine. With an elimination half-life of 8 to 30 hours, amoxapine is usually administered to adults at a dose of 200 to 300 mg daily. Amoxapine was reported to have comparable efficacy and faster onset at improving selected symptoms of depression, as compared with imipramine, and adverse events were less frequent (8). In our study, 20 patients treated with amoxapine reported semen ejaculation, a significantly higher success rate than that in patients receiving vitamin B_12_ (success rate: 80% vs 16%, respectively; P < 0.0001). We chose a daily amoxapine dose of 50 mg, after referring to a guideline for treating RE with imipramine. This dosage is about 25% of that normally used to treat depression and therefore would be expected to result in a low rate of adverse events. We observed no major adverse effects. Only 3 patients reported adverse events (mild sleepiness and constipation), which confirms that amoxapine at this dosage not only shows efficacy in treating RE but also is safe and well tolerated.

Another advantage of amoxapine administration is that it shows clinical benefit in the treatment of RE due to different causes. As Kamischke and Nieschlag noted in their review (12), neurogenic causes, such as pelvic surgery and diabetes mellitus, are responsible for a large number of RE cases. Among our patients, 60% had diabetes mellitus and 24% had undergone retroperitoneal lymph node dissection as part of surgical treatment for rectal carcinoma. These patients were clearly at high risk for RE due to sympathetic nerve impairment and thus were candidates for treatment with drugs that increase sympathetic tone. In our study, most men, including 1 patient with mild depression, successfully achieved antegrade ejaculation while receiving amoxapine. These results indicate that amoxapine can be used successfully to treat RE due to various causes. There was a total of 5 patients (20%) who did not respond to treatment with amoxapine. A possible explanation for the non-response to amoxapine was that the underlying pathology was severe or anatomic, i.e., that damage to organic structures could not be addressed by medical treatment alone.

It is clear that the bladder neck has an important role in normal ejaculation (13). Dysfunction of the autonomic nervous system and impairment of the internal urethral sphincter can inhibit bladder closure during expulsion of semen. The etiologies can be pharmacologic (e.g., use of an α-adrenoceptor blocker), anatomic (e.g., congenital abnormalities), neurogenic (e.g., retroperitoneal surgery, diabetic autonomic neuropathy or multiple sclerosis) and even idiopathic (14). As a result, semen can reflux into the bladder through the bladder neck, leading to total or partial absence of antegrade ejaculation despite the presence of orgasm (i.e., RE).

Pharmacotherapy for RE attempts to restore bladder neck function by either increasing sympathetic tone or decreasing parasympathetic activity (3). The medications used can be sympathomimetic, anticholinergic or antihistaminic and are recommended for men without spinal cord injuries or anatomic anomalies of the urethra who are not receiving medicines for other underlying illnesses (2). In a recent systematic review, the overall antegrade ejaculation rate was 28% (11/40) for sympathomimetic drugs, 22% (11/50) for anticholinergic drugs, and 39% (5/13) for the combination of sympathomimetics and anticholinergics (3). Although the efficacy of combination therapy seemed to be higher than that of mono-therapy, the numbers of patients in these studies were too small for any firm conclusions to be drawn.

Pharmacotherapy is more convenient, less invasive and more easily tolerated than other treatment methods for RE such as surgical management (15-17), electroejaculation (18) and urinary sperm retrieval (19-21). Moreover, most non-pharmacotherapy only addresses infertility and does not directly treat RE. Pharmacotherapy offers patients the possibility of resuming normal intercourse and natural pregnancy. The data in the present study showed that 56% (14/25) of the patients were unmarried and that 60% (15/25) did not require fertility treatment. To these patients, recovery of normal ejaculation may be more important than immediate improvement of fertility. Pharmacotherapy should be considered as the most appropriate first-line therapy for such patients.

Imipramine was found to be effective and safe for the treatment of RE due to anatomic and physiologic causes (4-7, 22, 23). However, the overall success rate did not exceed 50% (4-7). Although the success rates for treating RE due to post-retroperitoneal lymph node dissection were reported to be nearly 100% (22, 23), these studies lacked controls and the sample sizes were small. The adverse effects of imipramine were reported to be mild and included dizziness, weakness, palpitation, nausea and sweating. The overall rates of adverse effects in two studies were 45.45% (22) and 36.36% (7), respectively.

This study has several limitations. First, we were not able to include a placebo suitable for double blinding. Although vitamin B_12_ is commonly used for neuroprotection (24), it has to be given 3 times daily. Therefore, it cannot be excluded that the open-label design of the study may have introduced a degree of bias into the results. Second, the treatment outcomes were reported by the patients, which does not permit an objective evaluation of spermatozoa characteristics; this may have increased the likelihood of false positive results. However, an advantage of this method of assessment is that the patients were able to have sex and masturbate freely, without a timetable, and unnecessary nervousness and anxiety could therefore be avoided. Third, the study did not include an active control group such as imipramine, so it was not possible to compare the efficacy and safety of amoxapine with other drugs currently used to manage RE. Fourth, the sample size was relatively small for a randomized controlled trial. Since RE is uncommon, it is very difficult to recruit large numbers of patients. We enrolled as many patients as possible during a three-and-a-half-year period. Moreover, amoxapine is a tetracyclic antidepressant, and treatment for RE is off-label and without guideline support. Therefore, our study is just an exploratory study but not a confirmatory study. Since the difference in success rates between the amoxapine and vitamin B_12_ groups was large, our results can be considered reliable despite the small sample size. Future research should consider the effects of amoxapine on spermatozoa characteristics and compare amoxapine with imipramine or other drugs used to treat RE.

Our findings indicate that amoxapine has a significantly higher efficacy than vitamin B_12_ in the treatment of RE. Adverse events during treatment with amoxapine were mild and infrequent, and the drug was well tolerated. In summary, low-dose amoxapine may be a safe and effective drug for treating RE caused by a variety of reasons.

## ABBREVIATIONS

RE: Retrograde ejaculation
TCA: Tricyclic antidepressant agent
IIEF-5: International Index of Erectile Function
T: Testosterone
E2: Estradiol
LH: Luteinizing hormone
FSH: Follicle stimulating hormone
PRL: Prolactin
IVF: *in vitro* fertilization
ICSI: intracytoplasmic sperm injection
